# Frontal-subcortical defects correlate with task switching deficits in Parkinson’s disease

**DOI:** 10.17712/nsj.2017.3.20160572

**Published:** 2017-07

**Authors:** Amara Gul, Javed Yousaf, Hira Ahmad

**Affiliations:** *From the Department of Applied Psychology, The Islamia University of Bahawalpur, Bahawalpur, Punjab, Pakistan*

## Abstract

**Objectives:**

To examine correlation between frontal-subcortical/ parietal-cortical functioning and task switching.

**Methods:**

An experimental study was designed to examine objectives with 80 participants (40 patients with idiopathic Parkinson’s disease (PD) and 40 healthy controls). Patients were recruited from February until September 2016 at Recep Tayyip Erdogan Hospital Pakistan, Muzaffargarh, Pakistan and healthy controls participated from community. Participants were administered Parkinson’s disease cognitive rating scale and Word-Digit categorization task switching experiment.

**Results:**

In contrast to healthy controls, (i) PD patients showed impaired cognitive performance on frontal-subcortical and posterior-cortical functions as assessed through PD cognitive rating scale (ii) PD patients showed task switching deficits. Frontal-subcortical defects correlated with task switching deficits. Lesser the scores on frontal-subcortical functions, larger switch costs will appear. Frontal-subcortical defects significantly predicted task switch costs.

**Conclusion:**

Frontal-subcortical not the posterior-cortical dysfunctions are significant marker of task switching deficits.

Parkinson’s disease (PD) is a chronic degenerative disorder affecting over 9 million people in world’s most populous nations by 2030.^1^ Cognitive impairment (CI) is a recurrent and debilitating feature of PD. Studies using positron emission tomography (PET) showed reduced 18 Fluorodopa [18 F] uptake by the frontal cortex in PD as compared to healthy individuals. The influx constant in the frontal lobe was positively associated with memory task and executive strategies.^2^ Cognitive dysfunctions in PD are similar to those found in patients with frontal cortex lesions. There are 2 physiological mechanisms: deficient frontal dopamine and disturbance in corticostriatal circuits. Functional magnetic resonance imaging (fMRI) demonstrated decreased activation in regions of the prefrontal cortex (PFC) during performance of set-shifting task (Wisconsin card sorting test) in patients with PD as compared to healthy individuals. These PFC regions were coactivated with striatum in healthy controls.^3^ Fronto-striatal and prefrontal-collicular pathways are responsible for modulation of inhibitory control.^4^ Dysfunctions in these pathways influence the ability to suppress irrelevant stimuli during performance of attention demanding cognitive tasks such as stroop task switching paradigm. Patients with PD showed impaired switching due to depleted attentional resources.^5^ Structural MRI and Fdopa PET suggest that brain volume loss (PFC and hippocampal atrophy) and striatal dopaminergic depletion play a role in cognitive deficits.^6^ Cognitive impairment in PD patients ranges across several domains such as memory, attention and executive functions. Grey matter loss in frontal, temporal and frontal cortex, hippocampus and cholinergic structures correlate with CI.^8^ These neurochemical changes disturb rule-based shifting through deficits in rule generation, maintenance and rule selection processes.^9^ Frontal cortical areas modulate cognitive and motor processes. Left frontal cortex and dopaminergic transmission along nigrostriatal pathway is involved in executive control to overcome interference between task-sets.^10^ White matter and cholinergic pathway hyperintensities can predict deterioration in frontal lobe based cognitive functioning.^11^ Task switching is mainly a function of PFC. This paradigm involves rule-shifting and inhibitory control to minimize between task interference. Working memory is instrumental to recall task-based rules and perform accordingly. The present study was designed to compare frontal-subcortical/ posterior-cortical functioning and task switching performance between patients with PD and healthy individuals. Second objective was to assess correlation between frontal-subcortical/posterior-cortical functioning and task switching performance. Third objective was to examine the predictor of task switching deficits. It was hypothesized that (i) patients with PD would exhibit weaker frontal-subcortical and posterior cortical functioning as compared to healthy controls; (ii) PD patients would show task switching deficits, in contrast to healthy controls; (iii) frontal-subcortical functioning would correlate with task switching performance; and (iv) Frontal-subcortical defects could predict task switching deficits.

## Methods

### Sample

Eighty participants (40 patients with PD and 40 healthy controls (HC)) took part in the study (**Table 1**). Patients diagnosed with iodiopathic PD were recruited from Recep Tayyip Erdogan hospital, Muzaffargarh, Pakistan from February-September 2016. The inclusion criterion for patient group were as follows: (i) age 40-70 years; (ii) diagnosed as having idiopathic PD; and (iii) having dopaminergic antiparkinsonism drugs. Healthy controls were contacted through advertisement of the study in community with the inclusion criterion as: (i) age 40-70 years and (ii) no history/present symptoms of PD. The exclusion criterion for all participants: (i) neurological disorder such as epilepsy, stroke, Alzheimer’s disease, meningitis, head injury; (ii) color blindness/vision inaccuracy; (iii) dementia as assessed through Mini Mental Parkinson^12^ (cutoff ≤17/32) and (iv) depression assessed through Geriatric Depression Scale-short form13 (score >5). The study was designed as an experimental research.

**Table 1:** Demographic and clinical characteristics of the sample.

Variables	PD patients n=40*		HC n=40*			
	Mean±SD	Min.-Max.	Mean±SD	Min.-Max.	t	*p*-value
Age	57±8.00	(40-70)	58±9.00)	(40-70)	t (39)=0.92	0.36
Education (years)	10±1.94	(8-14)	11±2.01)	(8-14)	t (39)=0.85	0.39
PD duration (years)	7±2.00	(1-15)	-			
F-SF	48.90±12.36	(30-70)	108.67±2.96	(102-114)	t (39)=30.34	<0.001
P-CF	5.27±2.05	(2-10)	18.22±2.16	(10-20)	t (39)=30.68	<0.001

F-SF - Fronto-subcortical functions, P-CF - Posterior-cortical functions, PD - Parkinson's disease, HC - healthy controls,

* the ratio of the gender (m/f) was 20/20

### Instruments. Parkinson’s Disease Cognitive Rating Scale (PD-CRS)

The PD-CRS^14^ was designed to cover fronto-subcortical and posterior-cortical cognitive functions depending upon neural correlates as described in previous neuropsychological and neuroimaging studies. Fronto-subcortical cognitive functions cover working memory, attention, alternating, and action verbal fluencies, clock drawing, immediate and delayed verbal memory. Posterior-cortical cognitive functions include copy drawing of a clock and confrontation naming task. Scores on fronto-subcortical (0-114) and posterior-cortical functions (0-20) are computed by summing up the raw score on each corresponding sub-item. Higher scores shows better performance. The test has good psychometric characteristics.

### Word-Digit Categorization Task Switching Experiment

Task switching experiment was designed in E-prime software^15^ to examine switching abilities. Stimuli comprised of 16 images of a word and digit each. Tasks alternated every second trial according to the task switching paradigm.^10^ In word categorization task, the rule was to categorize the word as single vowel/double vowel word. In digit categorization, the rule was to categorize the digit as odd/even. Tasks were cued by different background colors. The experiment had 129 trials in total.

### Statistical Analyses

Differences on demographic and clinical data will be analyzed through paired sample t-test. Task switching data will be examined through repeated measures analysis of variance (ANOVA) with factors trial 2 (switch vs. repeat) x group 2 (PD vs. HC). Reaction times (RTs) for the first trial and those exceeding 2.5 standard deviation from each participant’s mean will be discarded. The RTs will be averaged across mean on switch and repeat trials (switch= 64 trials; repeat=64 trials). Switch costs will be calculated by subtracting mean RTs on repeat trials from mean RTs on switch trials). Switch costs reflect the speed of switching between 2 tasks. Higher switch costs show deficient switching ability.

### Procedure

The study was approved by ethic committee of the Islamia University of Bahawalpur, Bahawalpur, Pakistan and was conducted in accordance with the Helsinki declaration. All participants gave written informed consent. In a single testing session, participants completed PD-CRS and performed word-digit categorization task switching experiment. Upon completion, they were debriefed and thanked for their participation in the study.

## Results

Parkinson’s disease patients and HC were not statistically different on age, gender, and level of education. Patients with PD showed frontal-subcortical and posterior-cortical defects. In contrast, HC exhibited efficient performance on frontal-subcortical and posterior-cortical functions. Results from ANOVA showed significant main effects of trial F (1,78)= 1255.17, *p*<0.001, hp2=.94 and group F (1,78)= 195.46, *p*<0.001, hp2=.71. Repeat trials were faster than switch trials (757 vs. 1438 milliseconds). PD patients performed slower than HC (1261 vs. 934 milliseconds). The PD patients (M=810 milliseconds) showed task switching deficits (i.e., larger switch costs) in contrast to HC (M=498 milliseconds), t (39)= 8.75, *p*<0.001. The PD patients showed lower scores on frontal-subcortical t (39)= 30.34, *p*<0.001 and posterior-cortical functions t (39)= 30.68, *p*<0.001 as compared with HC. Bivariate correlation computation showed strong association between task switching deficits and frontal-subcortical functioning (r=-.70, *p*<0.001). There was no correlation between task switching deficits and posterior-cortical functioning (r=.11, *p*=.489). Linear regression analysis with predictors as frontal-subcortical and posterior-cortical functioning and task switching deficits as dependent factor proved significant model F (2, 39)=19.27, *p*<0.001, R^2^=0.51. Frontal-subcortical defects could predict task switching deficits b=-.70, t=6.13, *p*<0.001. Posterior-cortical defects failed to be the significant predictor of task switching deficits b=.09, t=0.80, *p*=.426.

## Discussion

The present study was designed to examine (i) task switching and frontal-subcortical/posterior cortical functioning in patients with PD (ii) correlation between task switching and frontal-subcortical/posterior cortical functions. Previous studies suggest that executive functions are modulated by frontal regions of the PFC. From neurobiological perspective, executive functioning is disturbed due to prefrontal atrophy, underactivation of ventral, lateral and dorsolateral regions of the PFC, hypometabolism of dopamine in dorsolateral PFC, white matter and dopaminergic pathway hyperintensities.^6^,^10^,^11^ Deficits in executive functions correlate with these physiological mechanisms. Frontal-subcortical and executive functioning rely on same brain regions and defects share similar etiological factors. There is a possibility that cognitive functioning in these areas correlate. Thus, the present study was designed to examine these correlations along with identification of predictors as frontal-subcortical and posterior cortical functioning. Results of the study showed a significant difference between PD patients and HC on PD-CRS and task switching. The PD patients showed impaired performance on both frontal-subcortical and posterior cortical functions. In contrast, HC showed efficient cognitive performance on frontal-subcortical and posterior cortical functions. Cognitive functions are deteriorated in patients with PD and appear at early stages of the disease. It was observed that patients with PD had difficulty concentrating on cognitive tasks that demand attention and execution of cognitive control to handle between task interference.^3^ Positron emission tomography scanning showed reduced 18 Fluorodopa uptake by the frontal cortex and caudate nucleus in brains of the patients with PD as compared with HC.^2^ These previous findings are consistent with task switching deficits observed in the present study. Patients with PD showed slower task switching performance as compared with HC. In addition, PD patients showed larger switch cost than HC. This result reflected their inability to overcome interference between tasks. On the contrary, HC were able to handle interference indexed as lesser switch cost. These cognitive dysfunctions are similar to patients with frontal cortex lesions. The fMRI studies provide support to these results which demonstrated weak co-activation in PFC and striatum in brains of PD patients.^5^ The interaction between frontal-striatal and prefrontal-collicular pathways exert cognitive control required to handle between task interference. Therefore, PD patients become deficient in inhibition and take longer time to perform attention demanding tasks.^4^ Further brain volume loss in PFC and hippocampus modulate CI.^6^ Grey matter is depleted in cortices along frontal, temporal regions and cholinergic structures causing impaired rule selection.^8^,^9^ In the present study, patients with PD showed longer switch costs as the task demands gets alternated. The participants on switch trials were required to perform according to the changed task rule. An inability to recall task demands resulting in larger switch costs. Task switching being an executive function correlated with frontal-subcortical functions. The results showed that frontal-subcortical defects are potential predictors of task switching deficits possibly because these 2 cognitive mechanisms are related and controlled by overlapping areas of the brain. Frontal-subcortical functions are comprised of executive functions, cognitive flexibility and memory tasks. Executive functions involved frontal regulation of interference, working memory and attentional processes. Dysfunctions in these cognitive areas are prominent in patients with PD. Switching task in the present study involved all components of frontal-subcortical functions. Task switch costs showed impaired frontal-subcortical functioning: (i) defective consolidation of working memory in order to recall task rules; (ii) cognitive inflexibility to shift between task-sets; (iii) resistance to task interference and (iv) dysregulation of attentional processes.

Findings of the present study has implication in identification of CI in PD specifically transition into dementia might be protected at early stages with task switching training to improve executive control. However, the present study did not explore social cognition. This factor might constitute a limitation since the possibility that PD could affect frontal-subcortical functions in the context of social cognition cannot be excluded. Future studies should examine whether switching deficits extend to social cognition and interfere with patient’s social communication.
